# Oral infections and orofacial pain in Alzheimer’s disease: Case report and review

**DOI:** 10.1590/S1980-57642010DN40200012

**Published:** 2010

**Authors:** Silvia Regina Dowgan T. de Siqueira, Thaís de Souza Rolim, Manoel Jacobsen Teixeira, Ricardo Nitrini, Renato Anghinah, José Tadeu T. de Siqueira

**Affiliations:** 1 DDS, PhD, Assistant Professor, Gerontology, School of Arts, Sciences and Humanities, University of São Paulo, São Paulo SP, Brazil.; 2 DDS, Postgraduate Student, Member of the Orofacial Team, Hospital das Clínicas, Medical School, University of São Paulo, São Paulo SP, Brazil.; 3 MD, PhD, Chairman of Neurosurgery, Neurology Department, Medical School, University of São Paulo, São Paulo SP, Brazil.; 4 MD, PhD, Associate Professor, Head of Group of Cognitive and Behavioral Medicine, Neurology Department, Hospital das Clínicas, Medical School, University of São Paulo, São Paulo SP, Brazil.; 5 MD, Group of Cognitive and Behavioral Medicine, Neurology Department, Hospital das Clínicas, Medical School, University of São Paulo, São Paulo SP, Brazil.; 6 DDS, PhD, Head of the Orofacial Pain Team, Dentistry Division, Medical School, University of São Paulo, São Paulo SP, Brazil.

**Keywords:** Alzheimer’s disease, elderly, dementia, temporomandibular disorder, orofacial pain

## Abstract

Dental infections, frequent in the general population, are a common cause of inflammation with systemic impact, and are the most common cause of orofacial pain. Temporomandibular disorders are also frequent in the elderly and represent an important cause of secondary headache. Both inflammation and pain can also contribute to cognitive, functional and behavioral impairment of the elderly and aggravate symptoms of patients with Alzheimer’s disease (AD). We report a case of a 74-year-old woman with AD and chronic facial pain who had a significant improvement in functional activities as well as in cognition and depressive symptoms after successful treatment of her facial pain. Patients with AD have higher compromise of oral health with infections and teeth loss. The investigation of orofacial pain should be performed in patients with AD, because of the associations reviewed and given the potential for improvement as highlighted by this case.

## Abstract (pt)

Infecções dentárias, frequentes na população e geral, são uma causa comum de inflamação com impacto sistêmico e a causa mais comum de dor orofacial. Disfunções temporomandibulares também são prevalentes nos idosos, causam cefaléia secundária. Tanto a dor quanto a inflamação podem também contribuir com o comprometimento cognitivo, funcional e comportamental em idosos e agravar sintomas em pacientes com doença de Alzheimer (DA). Este é um relato de um caso de uma mulher com 74 anos de idade com DA e dor orofacial crônica que apresentou uma melhora significante nas atividades funcionais assim como na cognição e sintomas depressivos depois do tratamento da dor facial. Pacientes com DA tem alto comprometimento da saúde oral com infecções e perdas dentárias. A investigação da dor orofacial deveria ser realizada em pacientes com DA devido às associações revisadas e à possibilidade de melhora como destacadas por este relato.

The aging process, or senescence, is characterized by gradual reduction in the functional reserve of the body, a phenomenon which compromises the ability to adapt to external and internal changes but is not an illness state.^1,2^ The elderly can be classified into three groups according to their general state of health: healthy (60% to 75% of elderly), chronically ill (20% to 35%) and fragile (2% to 10%).^1^ Among diverse symptoms that this group reports, pain can and must be evaluated as a potential sign of disease.^1^ Many elderly are edentulous, and oral infections are common in the adult population. At these ages, neurodegenerative diseases are also common, of which Alzheimer’s disease (AD) is the most prevalent.^3,4^ The objective of this article was to present a case and review the role of oral infections and orofacial pain in AD. Articles published between 1984 and 2008 were searched according to the following keywords: Alzheimer’s disease OR elderly OR dementia AND orofacial pain OR oral infections. A case reported in the literature is described below.

## Case report

A woman aged 74 years, diagnosed with mild to moderate AD^5^ an MMSE (Mini Mental State Exam)^6^ score of 18, and presenting severe functional impairment identified by a score of 30 on the Functional Activity Questionnaire (scores above 5 are indicative of functional impairment in dementia)^7^ had a complaint of left pre-auricular pain which spread to the head. The pain was throbbing and unbearable, with long crises (hours to days) 2-3 times a week, and had started 3 years earlier but had worsened in the last 10 months. She had little relief with common analgesics. She was completely edentulous but only using the superior prosthesis. Her masticatory muscles (temporalis, masseter and neck muscles) were painful on palpation on both sides. A dental exam was performed using the DMFT (decays, extractions and fillings), the OHIP (45) (assessing quality of life associated to oral health), and the Research Diagnostic Criteria for Temporomandibular Disorders (RDC/TMD) Axis I and II for musculoskeletal diagnosis of the masticatory system and psychosocial aspects involved (depression, anxiety and limitations in quality of life).

The proposed initial treatment was 4 applications of TENS (transcutaneous electric neural stimulation) to the masseter muscles, hot water compresses on the face and neck, and physiotherapeutic mouthwashes with 2% phenol water, three times a day. After 3 weeks, her pain had been alleviated and there was a marked improvement in the FAQ score, as well as in the MMSE and depressive symptoms (Table 1). She was referred for prosthesis treatment at the clinic, which was completed within 3 months. In the follow-up evaluation, she reported no pain or mandibular limitations.

**Table 1:** Cognitive, behavioral, functional, and mandibular characteristics of the patient before and after treatment.

	1^st^ Evaluation	2^nd^ Evaluation (after 3 weeks)
Pain (Visual Analogic Scale)	10	5
MMSE	18	21
FAQ	30	11
DMFT	32	32
OHIP14**	23.1	
Severe	8.5	
Good		
RDC/TMD Axis I	Myofacial pain without mouth opening limitation	No diagnosis
RDC/TMD Axis IIAnxiety	2.5Severe	1.0Severe
RDC/TMD Axis IIDepression	1.5Severe	0.85Moderate
RDC/TMD Axis IIJaw limitations	58%	0%

MMSE, Mini Mental State Examination; FAQ, Functional Activities Questionnaire; DMFT, Number of decayed, missing and filled teeth; OHIP 14, Oral Health Impact Profile.^45^ RDC/TMD Axis I and Axis II: Research Diagnostic Criteria for Temporomandibular Disorders, Axis I (clinical evaluation and TMD classification) and Axis II (psychosocial evaluation).

### Facial pain in the elderly

Pain was defined by the International Association for the Study of Pain (1986), as a sensorial and emotional unpleasant experience, associated to a real or potential tissue injury, or described in such terms.^8^ The denomination Orofacial Pain includes all painful conditions of the mouth and face, which are specific to dentistry, and including temporomandibular disorders (TMD).^8^ It s important to note that this is one of the areas of the whole body with the most types of diagnosis, often overlapping, and with significant psychological and social impact.^8,9^ Chronic pain in the community varies from 7% to 40%, and is persistent in 8% of individuals. In general, the most common cause is musculoskeletal, often diffuse.^10^

The perception of painful stimuli may be quantitatively and qualitatively modified by a series of internal and external factors^9,11^ where these include emotional state and awareness, social, cultural and environmental aspects, the meaning of pain experiences and finally, the context in which pain occurred. The first step in the sequence of events that give rise to the sensitive phenomenon of pain is the transformation of environmental stimuli into action potentials that are transmitted to the Central Nervous System (CNS) by the peripheral sensitive fibers.^12^ The activity of the nociceptors is modulated by chemical substances released in high concentrations in the tissue environment due to inflammatory, traumatic and/or ischemic processes.^13^ Pain is the most common symptom reported by the elderly and is present in more than 50% of their complaints; 19% of hospitalized elderly have moderate to severe pain. However, significant growth in the elderly population is expected in the next 50 years (groups more than 65 years old will grow from 7.4% to 16.4% and those older than 80 years old will triple by 2050 worldwide).^2,8^ In parallel with this increase in life expectation, quality of life and physical, psychological and social wellbeing is expected to be preserved at this age. Thus, pain should be identified and controlled.

In the elderly, pain is in general chronic and associated to degenerative and articular diseases, which occur in 50% to 70% of individuals between 60 and 85 years old. Headaches and facial pains are present in 3% to 5% of this group and completely edentulous patients are very common in the general population (particularly the third age), constituting an important cause of secondary headache largely due to TMD.^8^

### Dental infection and orofacial pain

Besides TMD, dental pain is an important cause of orofacial pain. Dental infections have an important role in the dentistry routine due to the risk of dissemination and given the severe complications that they can cause.^14^ The normal oral microorganism organization is very complex, and infection occurs with the imbalance between the commonly found microorganisms (which are the natural protection against throat and upper airway infections) and the pathogenic microorganisms under specific conditions.^14^

Such infections are generally not localized and in some circumstances affect bone tissue, muscles and mucosa spreading throughout the space of the fascias resulting in severe infection which can be extremely painful and even lead to mortality.^7^ The etiology of the oral septic process and the course of evolution of the infection will depend on the resistance of the organism and on the virulence of the pathogens.^7^

When the patient has a degree of immune compromise because of a primary pathology or a therapeutic situations (e.g. Transplants), dental infections carry higher risks due to immune suppression. Under these circumstances, patients can have higher dissemination of inflammation and infection, and more pain. It is important to bear in mind that the chemical mediators involved in periodontal inflammation are not only locally released by inflammatory cells but also by peripheral nerve endings causing neurogenic inflammation, usually diffuse and spread, which is very similar to masticatory muscle pain or atypical facial pain.^7,9^ In chronic patients with pain, periodontal disease should be investigated as a determinant factor as its treatment can improve the facial pain.

The release of bioproducts by the dental infection can activate the synthesis of prostaglandins and the release of bradykinin by plasmatic precursors. These, along with other mediators such as histamine from the mastocytes, act synergistically increasing edema through the accumulation of plasma fluid in the tissue. The inflammatory mediators excite and sensitize the peripheral nerve fibers resulting in spontaneous pain and in the increase of the pain sensation after a stimulus, characterizing hyperalgesia. The chemical mediators stimulate the release of neuropeptides by the nerve fibers.^9^. Recent studies demonstrate that substance P, the calcitonin gene related peptide (CGRP) and neurokinin A are higher in the gingival fluid in patients with irreversible pulpar pain compared to those with painless teeth, where these mediators are reduced after dental treatment.^15-16^ These neuropeptides are commonly found at the trigeminal ganglion^17^ and vary in the gingival fluid or saliva according to the presence of periodontal disease.^18,19^

Chronic infections are often associated to pain, especially in acutization. Among the possible causes, imbalance between the host and infection agent may be an underlying factor caused by increased virulence or weakness of the host. On the other hand, recent evidence shows that periodontal pain might occur even in chronic cases due to algogenic mediators in the tissue.^12,16-19^ There is also constant release of interleukins due to immune activation.^14,20-22^

Other diseases associated to the inflammatory process that can be worsened by dental infections are: rheumatic diseases, because of the pathogenic role of cytokines and interleukins;^23^ diabetes mellitus, given its association with higher prevalence of infections and the proinflammatory role of insulin;^24^ coronariopathies, owing to the inflammation role in vascular disease etiology,^25,26^ and also AD.

### Inflammatory activity and Alzheimer’s disease (AD)

There is evidence that inflammatory processes are relevant in the pathophysiology of AD ^27-30^ Cytokines represent more than 50 proteins that participate in the regulation of growth, development and activation of the immune system and in the mediation of the inflammatory response throughout the body.^23,24^ Patients with AD have high levels of interleukin 1 (IL-1), interleukin 6 (IL-6), and tumoral necrosis factor (TNF) in blood serum and in extracts of brain tissue. However, the exact role of the inflammatory process in AD is not known.^31-34^

### Dental infections and the Alzheimer’s disease (AD)

Many recent studies have shown that patients with AD have higher compromise of oral health with decays, infections and teeth loss.^30,35-39^ Among people with dementia, the prevalence of edentulism is higher than in the general population.^28,37,38^ There is also a reduction in masticatory efficiency proportional to cognitive impairment^29,40^ and it is noteworthy that cognitive impairment in patients with AD may contribute to the worsening of oral hygiene thus aggravating oral infections.^36,39,41^

Among dental infections, periodontal disease have being demonstrated as one of the most important in terms of activation of inflammation and systemic impact.^23^ Periodontal disease is defined as the infection and chronic inflammation of the supporting tissues of the teeth (gingival, periodontal ligament, alveolar bone) (American Academy of Periodontology - AAP, 1999). Many forms of periodontal disease can result in systemic inflammation which is characterized by increased serum C Reactive Protein, IL-1 and IL-6.^42^ Akin to other systemic diseases such as diabetes mellitus, rheumatoid arthritis and coronariopathies, periodontal disease can play a role in AD as an underlying chronic inflammation.^43^ Periodontal disease can predict higher serum C Reactive Protein concentration,^19^ where treatment of periodontal disease can reduce its concentration^21,23^.

On the other hand, these inflammatory factors (IL-1, IL-6, TNF, C Reactive Protein) are also increased in the blood of patients with AD^33,34^ and in elderly without AD but with cognitive impairment,^32^ and thus can be a marker for risk of developing dementia.^30^ In addition, infection can act directly by increasing the deposits of Aβ in patients with AD.^2^

### Pain relief and improvement of dementia symptoms

Dental infections and orofacial pain can play a role in cognitive, functional and behavioral impairments in AD as shown by the case of an individual with AD who had severe orofacial pain due to TMD and after TMD treatment and pain relief showed substantial improvement in emotional (anxiety and depression) aspects, quality of life and functional activities. The pain in this case was associated with a severe chronic headache, which was diagnosed as masticatory myofascial pain secondary to TMD.^44^ This type of TMD is characterized by diffuse, spreading, deep pain which is often related to the adjacent areas (head and neck). When evaluating a patient with AD, cognitive impairment represents only part of the signs and symptoms that can be affected, and this case reported an improvement in other parameters besides cognition, that contribute to dementia severity through the treatment of orofacial pain.

Dementia can impact mandibular function,^40^ and this might explain the development of TMD in this patient with AD. Moreover, the pain can aggravate function, quality of life and cognition.^11^ It is also known that the loss of posterior teeth can contribute to TMD,^10^ and that AD can contribute to teeth loss.^38^ The investigation of the causes of orofacial pain is crucial in patients with dementia given the associations reviewed and observed in this case.

To conclude, many studies have shown that individuals with cognitive impairment have more dental decay, worse oral hygiene and fewer teeth. Inflammatory activation by dental infections and its systemic implications can contribute toward modulating and aggravating the neurodegenerative process that occurs in AD. Long-term studies investigating the severity of dental infections, especially periodontal disease, along with its systemic interactions such as blood concentration of C Reactive protein and cytokines in patients with AD and healthy subjects should be conducted and future studies should investigate orofacial pain complaints to determine their exact participation and possible factors underlying these correlations. The role of masticatory function compromise in patients with AD should also be investigated.
